# GaN HEMT Oscillators with Buffers

**DOI:** 10.3390/mi16080869

**Published:** 2025-07-28

**Authors:** Sheng-Lyang Jang, Ching-Yen Huang, Tzu Chin Yang, Chien-Tang Lu

**Affiliations:** Department of Electronic Engineering, National Taiwan University of Science and Technology, Taipei 106335, Taiwan; m11119021@mail.ntust.edu.tw (C.-Y.H.); m10502337@mail.ntust.edu.tw (T.C.Y.); m10502316@mail.ntust.edu.tw (C.-T.L.)

**Keywords:** buffer, GaN HEMT power oscillator, figure of merit, left-handed transmission line, reliability, phase noise

## Abstract

With their superior switching speed, GaN high-electron-mobility transistors (HEMTs) enable high power density, reduce energy losses, and increase power efficiency in a wide range of applications, such as power electronics, due to their high breakdown voltage. GaN-HEMT devices are subject to long-term reliability due to the self-heating effect and lattice mismatch between the SiC substrate and the GaN. Depletion-mode GaN HEMTs are utilized for radio frequency applications, and this work investigates three wide-bandgap (WBG) GaN HEMT fixed-frequency oscillators with output buffers. The first GaN-on-SiC HEMT oscillator consists of an HEMT amplifier with an *LC* feedback network. With the supply voltage of 0.8 V, the single-ended GaN oscillator can generate a signal at 8.85 GHz, and it also supplies output power of 2.4 dBm with a buffer supply of 3.0 V. At 1 MHz frequency offset from the carrier, the phase noise is −124.8 dBc/Hz, and the figure of merit (FOM) of the oscillator is −199.8 dBc/Hz. After the previous study, the hot-carrier stressed RF performance of the GaN oscillator is studied, and the oscillator was subject to a drain supply of 8 V for a stressing step time equal to 30 min and measured at the supply voltage of 0.8 V after the step operation for performance benchmark. Stress study indicates the power oscillator with buffer is a good structure for a reliable structure by operating the oscillator core at low supply and the buffer at high supply. The second balanced oscillator can generate a differential signal. The feedback filter consists of a left-handed transmission-line *LC* network by cascading three unit cells. At a 1 MHz frequency offset from the carrier of 3.818 GHz, the phase noise is −131.73 dBc/Hz, and the FOM of the 2nd oscillator is −188.4 dBc/Hz. High supply voltage operation shows phase noise degradation. The third GaN cross-coupled VCO uses 8-shaped inductors. The VCO uses a pair of drain inductors to improve the *Q*-factor of the *LC* tank, and it uses 8-shaped inductors for magnetic coupling noise suppression. At the VCO-core supply of 1.3 V and high buffer supply, the FOM at 6.397 GHz is −190.09 dBc/Hz. This work enhances the design techniques for reliable GaN HEMT oscillators and knowledge to design high-performance circuits.

## 1. Introduction

A Gallium Nitride (GaN) High Electron-Mobility Transistor (HEMT) has emerged as a potential high-power and high-speed transistor for commercial and military applications due to its superior material performance, such as a large bandgap, high saturation and peak velocity, and good thermal conductivity. GaN HEMTs offer comparable noise levels to conventional GaAs HEMT devices [1] and better noise figure compared to bipolar SiGe HBT and InGaP HBT [2]. A low-phase-noise oscillator is a key component of many radio-frequency communication systems, and the reliability of the oscillator helps the design of GaN HEMT circuits for high-power applications.

GaN HEMT oscillators can be designed with a GaN HEMT amplifier with a feedback network [3,4]. With the feedback method, the power amplifier and the oscillator are combined in one for low power consumption, and no buffer is used. But the output matching network may degrade the oscillator’s performance. On the other hand, an oscillator and a buffer form a composite high-power oscillator. The role of the buffer is to duplicate the low-phase signal from the core oscillator and provide large output power. The oscillator can use different supply voltages for the VCO core and the buffer for performance optimization. This paper studies three GaN HEMT oscillators with HEMT buffers. The first 0.25 μm GaN HEMT oscillator consists of a split core and a buffer, demonstrating the advantage of this design from the reliability point of view. The core oscillator is used to improve the phase noise at low supply voltage with high reliability.

Balanced oscillators [5,6] based on Colpitts oscillator topology have good phase noise performance. Secondly, this paper describes a balanced 0.25 μm GaN [7] oscillator with a left-handed transmission-line *LC* network as the high-pass feedback filter, which provides impedance matching and delay phase control. Cross-coupled GaN HEMT oscillators [8,9,10] are made of two HEMTs; the differential topology suppresses the substrate coupling effect. This third cross-coupled 0.12 μm GaN oscillator, embedded with an implicit harmonic *LC* network, improves the phase noise, and it uses 8-shaped inductors to suppress the magnetic coupling noise.

## 2. X-Band Feedback GaN HEMT Oscillator with Split Core and Buffer

### 2.1. Circuit Design of the First Oscillator

**Figure 1:**
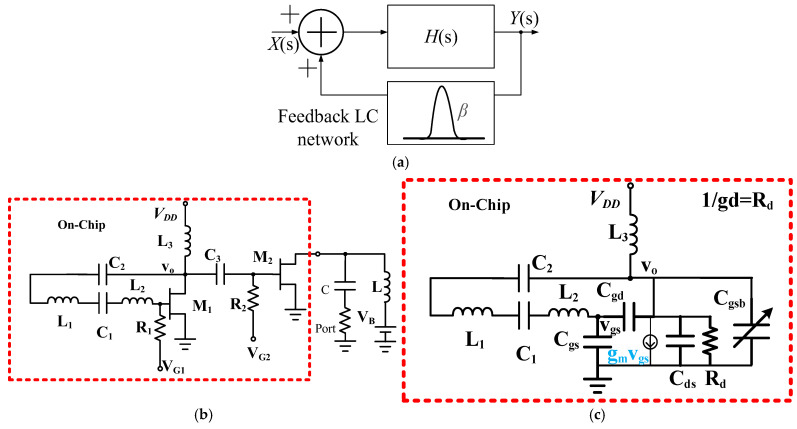
(**a**) Block diagram of a feedback oscillator with an amplifier *H*. (**b**) Schematic and (**c**) equivalent circuit of the first HEMT oscillator.

Figure 1a shows a feedback oscillator generating a periodic output *Y* without input *X* = 0. The feedback network circuit *β* is used to select the operation frequency. The circuit can oscillate if Barkhausen’s criteria |*βH*(jω_o_)| = 1 and ∠*βH*(jω_o_) = 0° hold. Figure 1b shows the schematic of the designed single-ended GaN HEMT oscillator. It consists of an oscillator and a buffer. The inductor *L*_3_ and HEMT *M*_1_ form an amplifier, and *R*_1_ is a gate-biasing resistor, and *V_G_*_1_ is the gate voltage. Inductors *L*_1_ and *L*_2_, capacitors *C*_1_ and *C*_2_*_,_* and the parasitic gate-source capacitor of *M*_1_ form the feedback filter. *V*_DD_ is the supply voltage and is connected to the output node of *L*_3_. The oscillator is used to drive the following buffer amplifier *M*_2_. HEMT *M*_2_ forms a buffer amplifier with gate bias at *V_G_*_2_ through the gate-bias resistor R_2_. *V*_B_ is the buffer drain bias through an external bias inductor *L*. With different *V*_DD_ and *V*_B_, the oscillator can be optimized for low phase noise performance, and the buffer is used to output high power. *M*_1_ and *M*_2_ share the same substrate stray inductance; the current of *M*_2_ affects the property of *M*_1_. Figure 1c is the simplified small-signal equivalent circuit of the designed HEMT oscillator. *C*_gsb_ is the parasitic gate-source capacitor of buffer *M*_2_. *C*_gs_, *C*_ds_, and *C*_gd_ are, respectively, the gate-source, drain-source, and gate-drain capacitors of *M*_1_. These capacitors are bias-dependent. *g_m_* is the transconductance of *M*_1_, and *g_d_* is the output conductance due to the effect of *M*_1_ output conductance and other resistive losses. *C*_3_ is ignored for simplicity. Minimum transconductance gain is given by

(1) $$g_{m}=\frac{\omega^{2}L_{21}C_{x-1}}{C_{x}/C_{gs}}g_{d}$$

(2) $$\frac{1}{C_{x}}=\frac{1}{C_{gs}}+\frac{1}{C_{1}}+\frac{1}{C_{2}},\;L_{21}=L_{2}+L_{1}$$

The small-signal model for the oscillator is used to derive the resonant frequency given by

(3) $$\omega^{2}=\frac{C_{x}L_{3}+L_{3}C_{gsb}+L_{21}C_{x}}{{2L}_{3}C_{gsb}L_{21}C_{x}}+\frac{\sqrt{{\left(C_{x}L_{3}+L_{3}C_{gsb}+L_{21}C_{x}\right)}^{2}-4L_{3}C_{gsb}L_{21}C_{x}}}{{2L}_{3}C_{gsb}L_{21}C_{x}}$$

As *V_DD_* increases, the voltage swing of *V*_o_ increases, and the average capacitance over an oscillation cycle changes. DC bias also changes the capacitance. The oscillation frequency is tunable by tuning *V*_DD_. If *L*_3_ is large, (3) simplifies to

(4) $$\omega =\sqrt{\frac{1}{L_{21}}\frac{C_{x}+C_{gsb}}{C_{gsb}C_{x}}}$$

### 2.2. Measurement and Discussion

**Figure 2:**
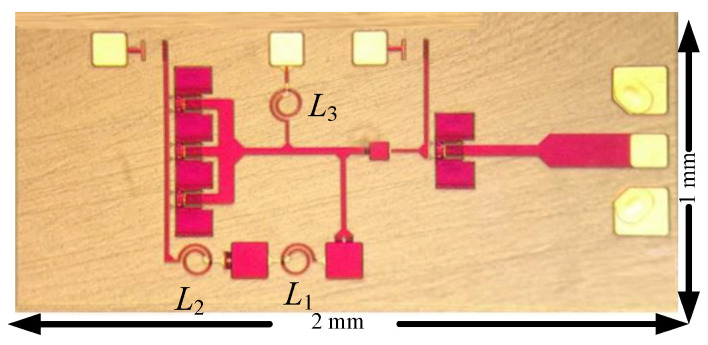
Chip micrograph for the HEMT oscillator. 2 mm × 1 mm.

**Figure 3:**
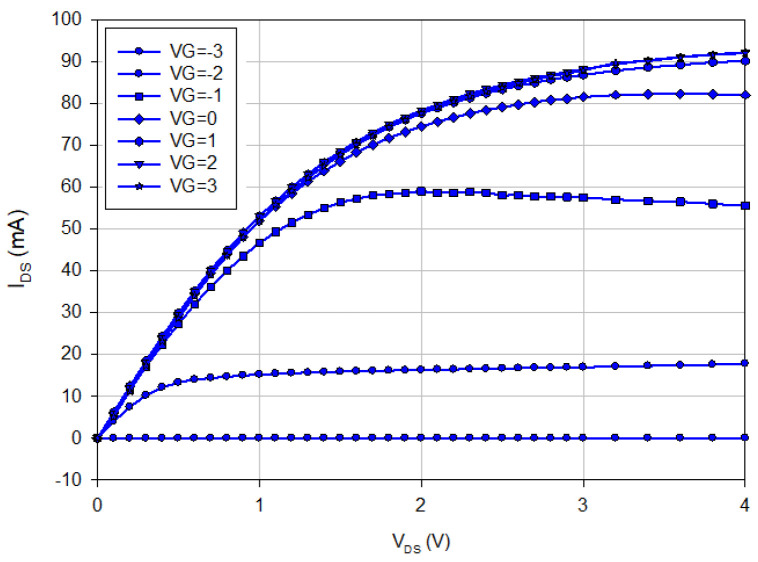
Measured I-V curve of buffer HEMT. Size: L = 0.25 μm, W = 75 μm, Number of fingers = 2.

**Figure 4:**
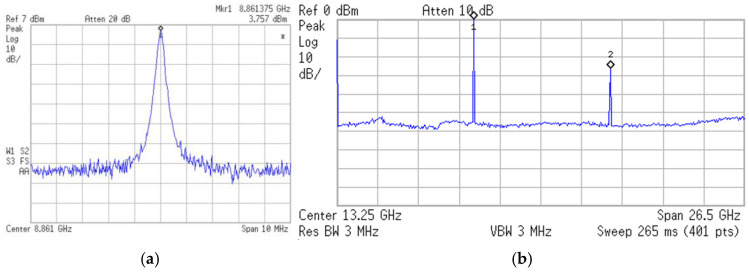
Measured (**a**) expanded spectrum and (**b**) full-span spectrum. *V*_DD_ = 0.8 V, *V*_G1_ = −2.4 V, *V*_B_ = 3 V, and *V*_G2_ = −2 V.

The first oscillators were designed and fabricated in the WIN 0.25 μm GaN/SiC HEMT process. Figure 2 shows the micrograph of the oscillator with a chip area of 2 × 1 mm^2^, including all test pads and dummy metal. Foundry-supplied inductors are planar circular inductors featuring a high-*Q* factor. Figure 3 shows the measured output characteristics of the buffer HEMT. Figure 4a shows the measured expanded output spectrum. Figure 4b shows the measured full-span output spectrum of the fundamental signal at 8.86 GHz with an output power of 3.75 dBm. At *V*_DD_ = 0.8 V, the power consumption is 2.45 mW. The 2nd harmonic power is lower than the fundamental by 30.3 dB.

**Figure 5:**
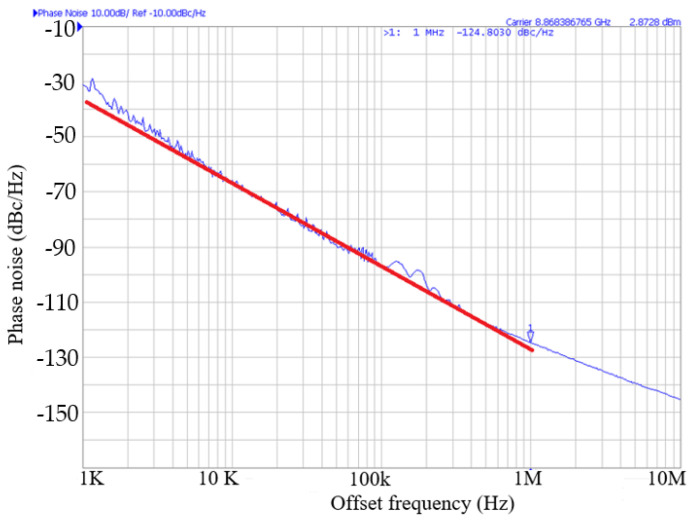
Measured phase noise. *V*_DD_ = 0.8 V, *V*_G1_ = −2.4 V, *V*_B_ = 3 V, and *V*_G2_ = −2 V. A red 1/*f* ^3^ guideline is used for reference.

**Figure 6:**
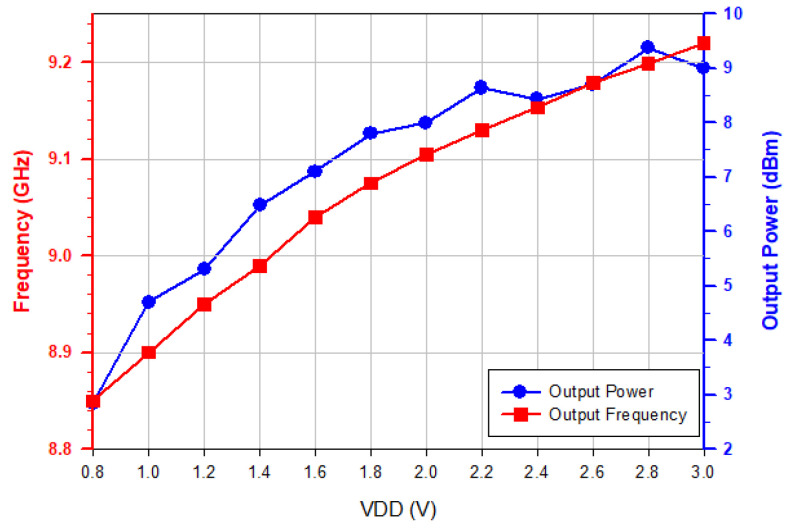
Measured output power and frequency versus *V*_DD_. *V*_G1_ = −2.4 V, *V*_B_ = 3 V, and *V*_G2_ = −2 V.

Figure 5 shows the measured phase noise performance of the oscillator. The phase noise of the oscillator is about −124.8 dBc/Hz at 1 MHz offset frequency from 8.86 GHz oscillation frequency and has a slope of −30 dB/dec in the low frequency offset. The phase noise is mainly due to the upconversion of device flicker current noise. Figure 6 shows the measured oscillator frequency and output power. As *V*_DD_ increases, the output power increases, the capacitance decreases, and the oscillation frequency *f_osc_* increases.

The figure of merit (FoM) defined in Equation (5) is used to benchmark the performance of oscillators:

(5) $$\mathrm{FOM}=L(\omega_{o},\Delta \omega )+10\cdot \log\left(P_{DC}\right)-20\cdot \log\left(\frac{\omega_{o}}{\Delta \omega }\right)$$

where ω_o_ is the fundamental frequency, ∆ω is the offset frequency, *L* {∆ω} is the phase noise at ∆ω, and *P_DC_* is the DC power consumption of the oscillator in mW. By the calculation, the FoM of the first oscillator is −199.8 dBc/Hz.

**Figure 7:**
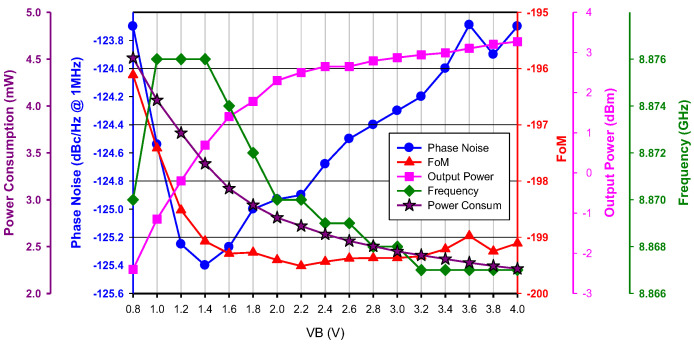
Measured buffer bias effect. *V*_DD_ = 0.8 V, *V*_G1_ = −2.4 V, *V*_B_ = 0.8~4 V, and *V*_G2_ = −2 V.

Figure 7 shows the effect of buffer bias *V*_B_ on oscillator performance. As *V*_B_ increases, output power from the buffer increases, and power dissipation in the oscillator core decreases. Oscillation frequency is insensitive to *V*_B_, and minimum phase noise is measured at *V*_B_ = 1.4 V. Considering the effect of buffer power consumption, a new FOM is defined by

(6) $${\mathrm{FOM}}_{p}=L(\Delta \omega )+10\cdot \log\left(\frac{P_{core}+P_{buffer}}{P_{out}}\right)-20\cdot \log\left(\frac{\omega_{o}}{\Delta \omega }\right)$$

**Figure 8:**
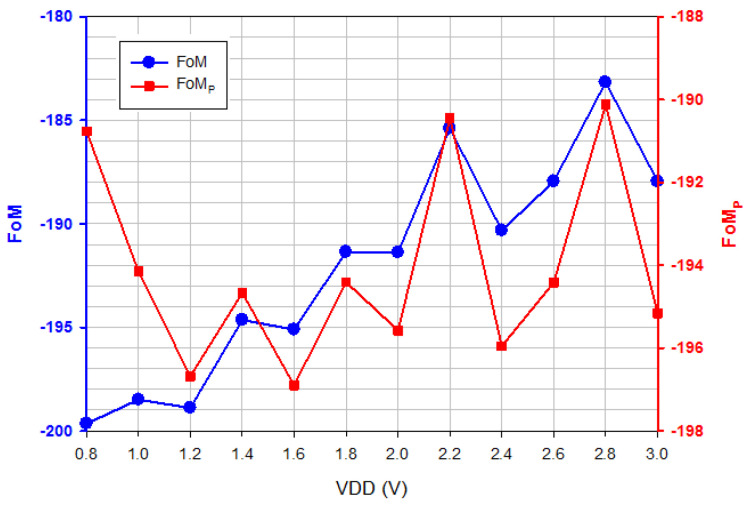
FOM and FOM_p_ versus *V*_DD_. *V*_DD_ = 0.8~3 V, *V*_G1_ = −2.4 V, *V*_B_ = 3 V, and *V*_G2_ = −2 V.

*P_OUT_* is the output power of the buffer, and *P_Buffer_* is the buffer power consumption. Figure 8 shows a comparison of FOM and FOMp versus *V*_DD_. FOM decreases with V_DD_, and FOM_P_ is relatively insensitive to V_DD_ because *P_Core_* is smaller than *P_Buffer_*. The phase noise of the oscillator is due to the AM-PM up-conversion caused by the device’s current noise and voltage-dependent capacitance. Table 1 shows the performance comparison. A GaN HEMT has deep-level defects located on the surface, barrier, or buffer layer of the device; the presence of deep-level defects could contribute to low-frequency current noise. It was found that the low-frequency current noise of GaN HEMT [11,12] is dominated by the flicker noise. The low-frequency current noise of *M*_1_ flows into *C_gs_*, *C_ds_*, and *C_gd_*, changes the capacitor voltage, and causes the fluctuation of average capacitance and oscillation frequency [13].

### 2.3. Drift of GaN HEMT Oscillator Performance with High Operating Bias

**Figure 9:**
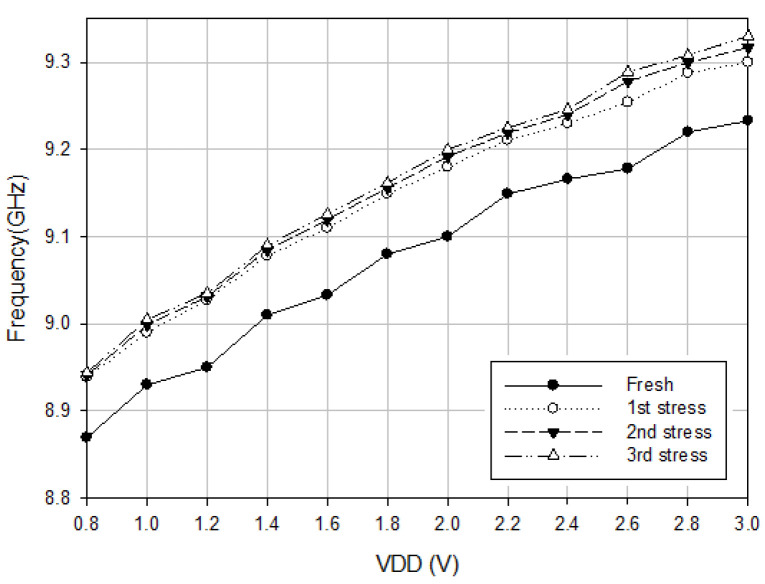
Measured oscillation frequency versus *V*_DD_. *V*_B_ = 3 V, *V*_G1_ = −2.4, and *V*_G2_ = −2 V.

High-power GaN HEMT oscillators operate the HEMTs in the high field with the hot-carrier stress causing the electron trapping and the creation of interface states, which increase the phase noise of the oscillator. In this subsection, the degradation of the oscillator with HEMTs subjected to hot-carrier injection is discussed. The stressed oscillator is subject to higher drain voltage operation: *V*_DD_ = 8 V, *V*_G1_ = −2.4 V, *V*_G2_ = 0 V, *V*_B_ = 0 V for 90 min, which is divided into three time steps classified at t = 0, 30, 60, and 90 min for measuring the time-dependent oscillator performance at low supply voltage while the buffer is on. Figure 9 shows the measured oscillation frequency versus *V*_DD_, and the oscillation frequency increases with *V*_DD_. The stress increases the oscillation frequency. The oscillation frequency is related to the tank capacitance; the stress increases the oscillation frequency, and a high supply voltage bias reduces the capacitance.

**Figure 10:**
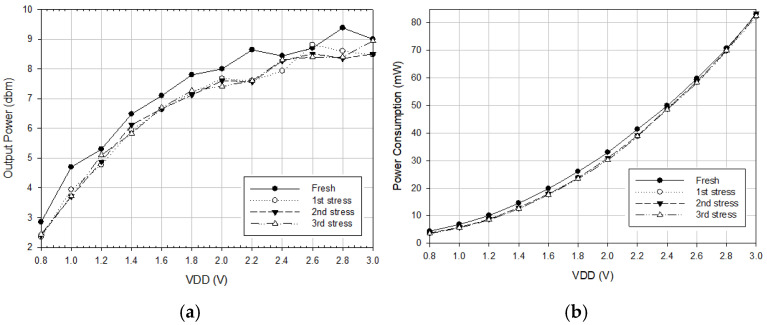
(**a**) Measured output power versus *V*_DD_. (**b**) Measured power consumption versus *V*_DD_. *V*_B_ = 3 V, *V*_G1_ = −2.4, and *V*_G2_ = −2 V.

**Figure 11:**
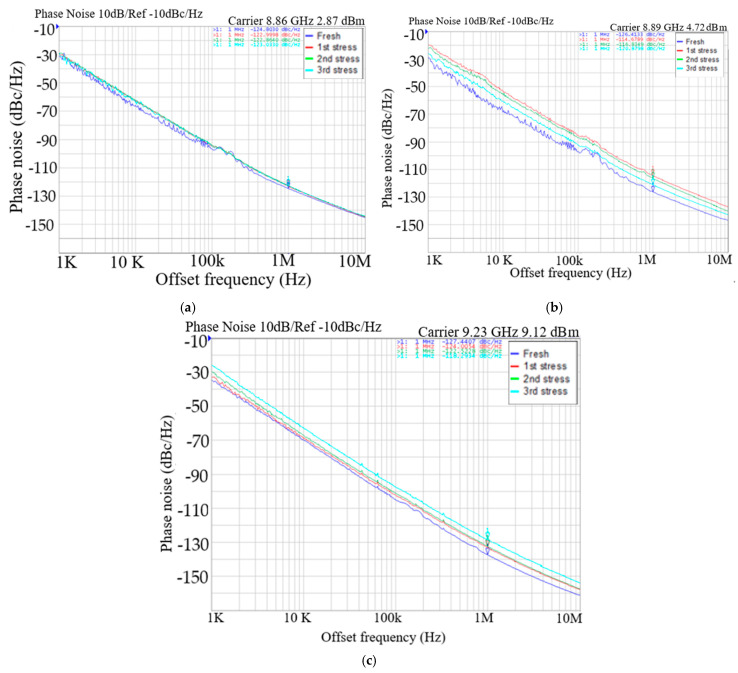
Measured phase noise versus step stress. (**a**) *V*_DD_ = 0.8 V, *V*_B_ = 3 V, *V*_G1_ = −2.4, and *V*_G2_ = −2 V. Measured on chip 1. (**b**) *V*_DD_ = 1 V, *V*_B_ = 3 V, *V*_G1_ = −2.4, *V*_G2_ = −2 V. (**c**) *V*_DD_ = 3 V, *V*_B_ = 3 V, *V*_G1_ = −2.4, and *V*_G2_ = −2 V.

**Figure 12:**
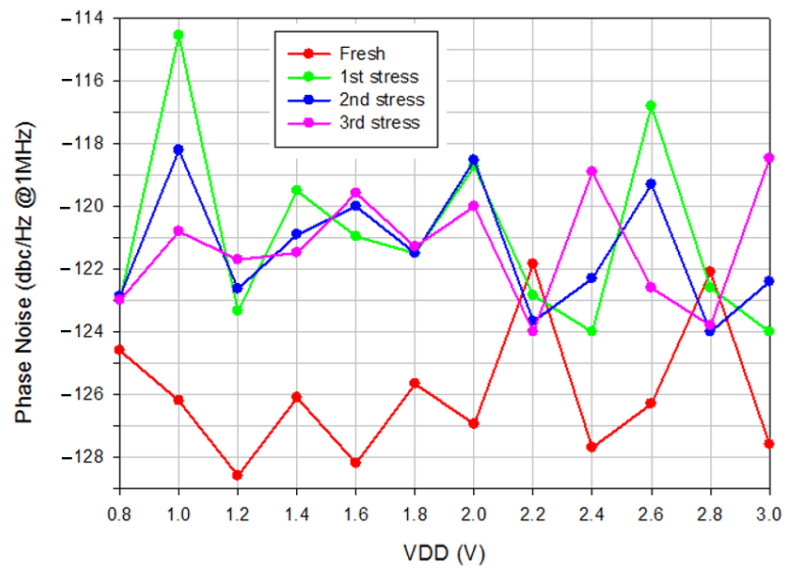
Measured phase noise at 1 MHz offset frequency versus *V*_DD_. *V*_B_ = 3 V, *V*_G1_ = −2.4, and V_G2_ = −2 V. Measured on chip 1.

**Figure 13:**
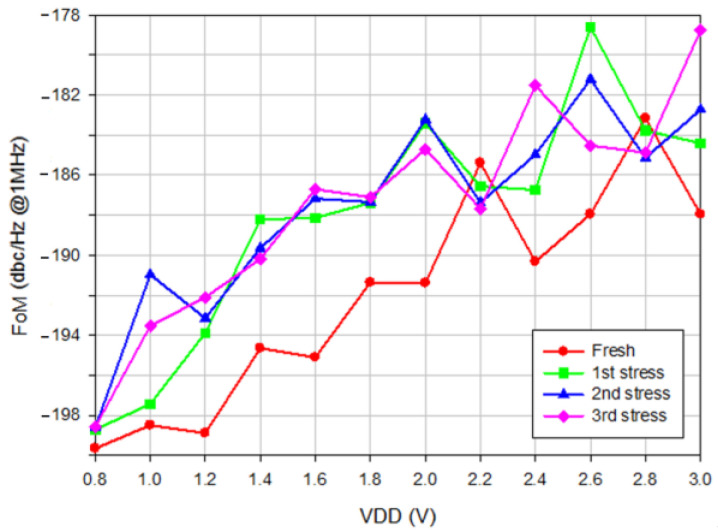
Calculated FOM. *V*_B_ = 3 V, *V*_G1_ = −2.4, and *V*_G2_ = −2 V. Measured on chip 1.

Figure 10a shows the measured oscillator output power versus *V*_DD_, where the *V*_DD_ increases the output power. Figure 10b shows measured power consumption versus *V*_DD_. The stress reduces the oscillator’s output power and power consumption. Figure 11a, Figure 11b, and Figure 11c show, respectively, the measured phase noise versus stress step at *V*_DD_ = 0.8, 1, and 3 V, indicating that the fresh phase noise is the smallest at low and high offset frequencies. Figure 12 shows measured phase noise at 1 MHz offset frequency versus *V*_DD_. At *V*_DD_ = 0.8 V, *V*_G1_ = −2.4 V, *V*_B_ = 3 V, *V*_G2_ = −2 V, the measured fresh phase noise of the oscillator is about −124.8 dBc/Hz at 1 MHz offset frequency from the carrier frequency 8.86 GHz. Figure 13 shows the FOM versus *V*_DD_. The high drain bias increases phase noise and degrades the FOM at *V*_DD_ < 2 V.

**Figure 14:**
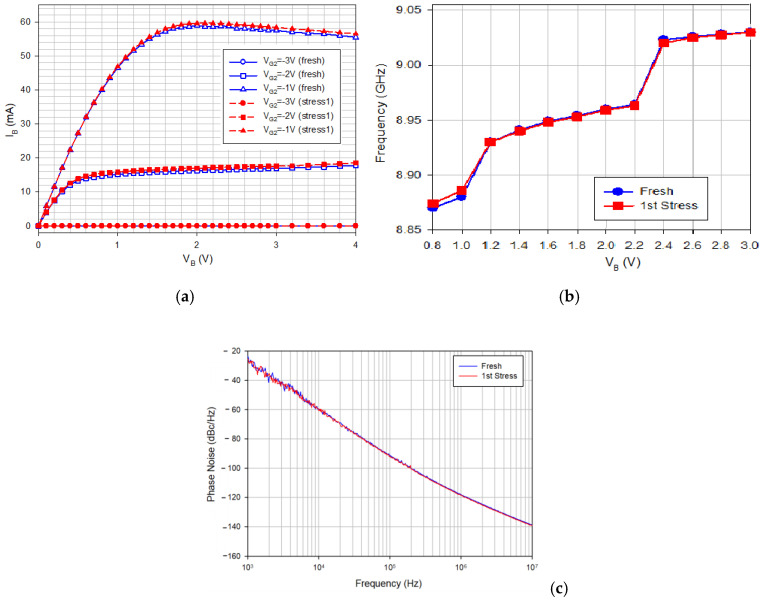
(**a**) Measured fresh and stressed I-V of buffer FET versus *V*_B_. Measured on chip 2. *V*_G2_ = −1, −2, and −3 V. Blue: fresh, and red: post-stress. (**b**). Measured oscillation frequency versus *V*_B_. *V*_DD_ = 0.75 V, *V*_G1_ = −2.4, and *V*_G2_ = −2 V. Measured on chip 2. (**c**) Measured phase noise versus step stress. *V*_DD_ = 0.75 V, *V*_B_ = 2 V, *V*_G1_ = −2.4, and *V*_G2_ = −2 V. Measured on chip 2.

For the next stress experiment, the buffer is subjected to higher drain voltage operation: *V*_G1_ = 0 V, *V*_DD_ = 0 V, *V*_G2_ = −2.2 V, and *V*_B_ = 8 V for 30 min, and the VCO-core is under low supply voltage. Figure 14a shows the measured output characteristics of buffer HEMT before and after the high buffer bias. Figure 14b shows measured oscillation frequency versus *V*_B_, and Figure 14c shows measured phase noises versus step stress. The buffer stress alone does not change the oscillation frequency and phase noise of the oscillator under low supply bias. This indicates that biasing the oscillator core at low supply and the buffer at high supply voltage is a good operation condition for large output power because high FOM is maintained. To gain high output power, the buffer supply voltage is increased.

## 3. Balanced GaN HEMT Oscillator with Left-Handed Transmission Line Filter

### 3.1. Circuit Design of the Balanced Oscillator

**Figure 15:**
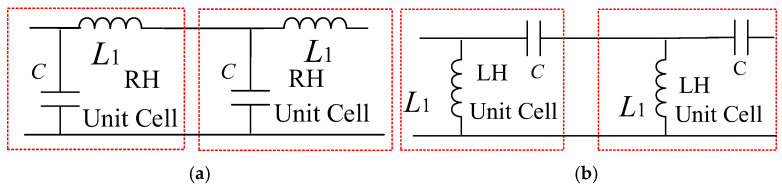
(**a**) Right-handed T-lines and (**b**) left-handed T-lines.

The previous oscillator is a single-ended circuit, and this section discusses a balanced oscillator using two identical single-ended oscillators configured in a balanced structure. In the balanced oscillator, the feedback network can be a lossless transmission line (TL) such as a right-handed TL (RH-TL) consisting of a series inductor *L*_1_ and parallel capacitor *C*, as shown in Figure 15a, or a left-handed TL (LH-TL) consisting of a shunt inductor *L*_1_ and series capacitor *C*, as shown in Figure 15b; the composite right/left-handed TL (CRLH-TL) is a general TL with a combination of the RH-TL and LH-TL. The LH-TL [14] and CRLH-TL [15,16] have been used to design CMOS cross-coupled oscillators. The present GaN HEMT balanced oscillator employs the LH-TL as the feedback network by cascading three LH-TL unit cells.

**Figure 16:**
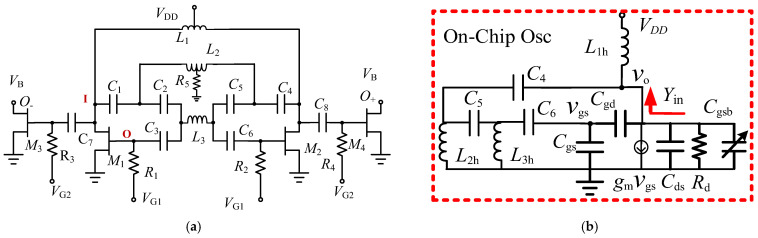
Schematic (**a**) and equivalent circuit (**b**) of the second oscillator.

Figure 16a shows the schematic of the designed fixed-frequency GaN HEMT oscillator. It consists of two single-ended sub-oscillators sharing the same virtual ground. The first sub-oscillator consists of a feedback filter and an amplifier formed with inductor *L*_1_ and HEMT *M*_1_. *R*_1_ is a gate-biasing resistor, and *V*_G1_ is the gate voltage for *M*_1_. Inductors *L*_1_, *L*_2_, and *L*_3_, capacitors *C*_1_, *C*_2_, and *C*_3_ form a left-handed filter for feedback from the output at the I-node to the input of the amplifier at the O-node. The circuit of a standing wave oscillator (SWO) uses an open-ended TL. The filter acts as an impedance transformer between the input and output of the amplifier, and it also provides a phase response to set the oscillation frequency. *V*_DD_ is the supply voltage. The second sub-oscillator consists of the same components as the first sub-oscillator. The common node of *L*_2_ and *R*_5_ is a virtual ground for the differential mode signals, and the common mode signal will develop a voltage at this node. *R*_5_ is the resistor used to diminish the common-mode oscillation. HEMT *M*_3_ and *M*_4_ forms buffer amplifiers with gate bias at *V_G_*_2_ through the gate-bias resistors *R*_3_ and *R*_4_. *V*_B_ is the buffer drain bias through an external bias inductor *L*. The I-port shows the largest voltage swing, and the O-port shows the smallest voltage swing; both ports show opposite voltage phases. C_1_ and *L*_2_ cause a phase shift and decrease the voltage swing.

In Figure 16b, a single-ended equivalent small-signal circuit for the designed oscillator is shown. *C*_gs_, *C*_ds_, and *C*_gd_ are, respectively, the gate-source, drain-source, and gate-drain capacitors of *M*_2_. *C*_gsb_ is the capacitor owing to *C*_8_ in series with the gate-source capacitor of *M*_4_. These parasitic capacitors are bias-dependent. Device flicker noise and thermal noise are up-converted to oscillator phase noise through these capacitors via the AM-PM modulation. *g*_m_ is the transconductance of *M*_2_, and *g*_d_ is the output conductance due to the effect of *M*_2_ output conductance and other resistive losses. The input impedance *Y*_in_ shows the effect of a high-pass 6th–order resonator. If *C*_gd_ is ignored, then *Y*_in_ is given by

(7) $$Y_{in}=\frac{den}{{\mathrm{sL}}_{1h}num}$$

(8) $$num=sC_{4x}{\mathrm{sL}}_{2h}\{1+s^{2}L_{3h}{[C}_{6x}+C_{5x}]\}+1+s^{2}{[L}_{3h}C_{6x}+L_{3h}C_{5x}+L_{2h}C_{5x}]+s^{4}L_{2h}C_{5x}L_{3h}C_{6x}]$$

(9) $$\begin{array}{l} den=1+s^{6}L_{1h}C_{4x}L_{2h}C_{5x}L_{3h}C_{6x}+s^{2}{[L}_{3h}C_{6x}+L_{3h}C_{5x}+L_{2h}C_{5x}+C_{4x}L_{2h}+L_{1h}C_{4x}]\\ +s^{4}{\{L}_{2h}C_{5x}L_{3h}C_{6x}+C_{4x}L_{2h}L_{3h}{[C}_{6x}+C_{5x}]+L_{1h}C_{4x}{[L}_{3h}C_{6x}+L_{3h}C_{5x}+L_{2h}C_{5x}]\} \end{array}$$

If *C*_5_ and *L*_2h_ are removed, the input admittance of the 4th-order resonator becomes

(10) $$Y_{in}=\frac{1+s^{2}{[L}_{3h}C_{6x}+L_{3h}C_{4x}+L_{1h}C_{4x}]+s^{4}L_{1h}C_{4x}L_{3h}C_{6x}}{{\mathrm{sL}}_{1h}\{1+s^{2}L_{3h}{[C}_{6x}+C_{4x}]\}}$$

The resonant frequency is obtained by setting *Y*_in_ = 0.

(11) $$\omega^{2}=\frac{L_{3h}C_{6x}+L_{3h}C_{4x}+L_{1h}C_{4x}}{2L_{1h}C_{4x}L_{3h}C_{6x}}\pm \frac{\sqrt{{{[L}_{3h}C_{6x}+L_{3h}C_{4x}+L_{1h}C_{4x}]}^{2}-4L_{1h}C_{4x}L_{3h}C_{6x}}}{2L_{1h}C_{4x}L_{3h}C_{6x}}$$

**Figure 17:**
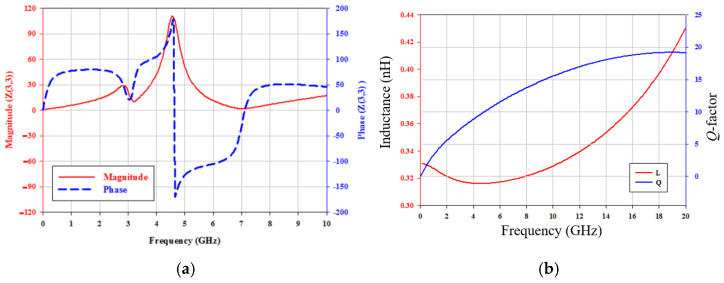
(**a**) Simulated input impedance at the drain of *M*_1_. *V*_DD_ = 2 V, *V*_G1_ = −2 V, *V*_B_ = 2 V, and *V*_G2_ = −2 V. (**b**) Simulated inductance and *Q*-factor of inductor *L*_1_ (= *L*_2_ = *L*_3_).

The oscillation frequency should obey the Barkhausen criteria. Figure 17a shows the simulated input impedance at the drain of *M*_1_, with a peak resonant frequency of 4.56 GHz. The second resonant frequency is 2.9 GHz, and the third resonant frequency is out of the *x*-axis plot. Figure 17b shows the simulated inductance and *Q*-factor of the inductor *L*_1_. The inductance is 0.316 nH, and the *Q*-factor is 9.643 at 4.56 GHz.

### 3.2. Measurement and Discussion

**Figure 18:**
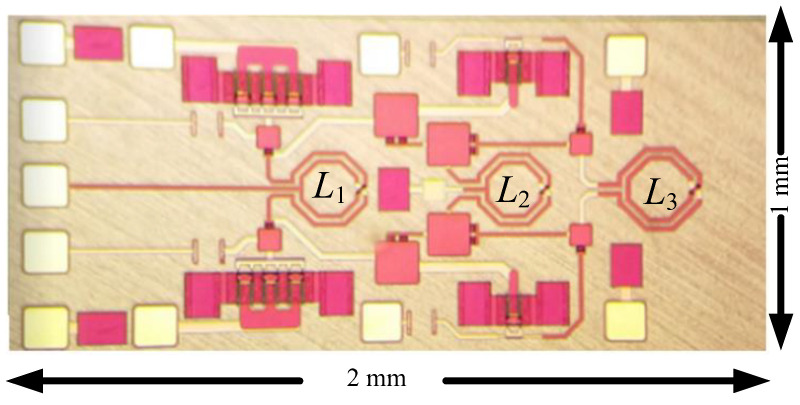
Chip micrograph for the HEMT oscillator. 2 mm × 1 mm.

**Figure 19:**
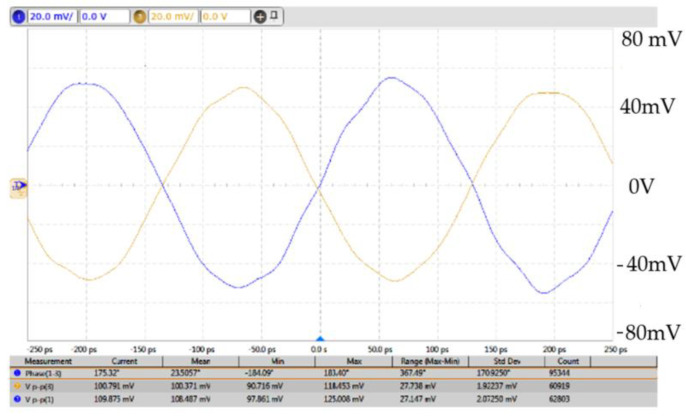
Measured output voltages. *V*_DD_ = 0.6 V, *V*_G1_ = −2.1 V, *V*_B_ = 0.8 V, and *V*_G2_ = −2.1 V.

**Figure 20:**
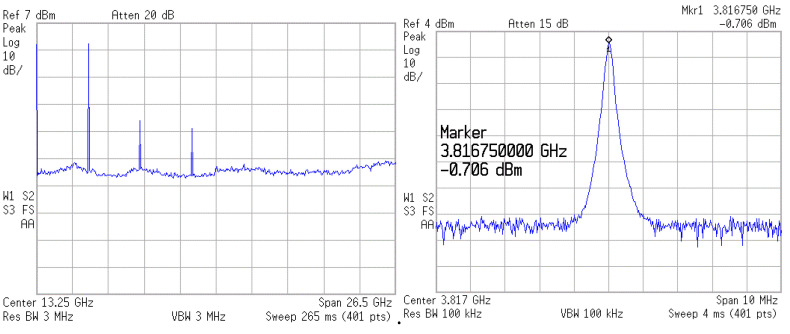
Measured spectrum. *V*_DD_ = 1.4 V, *V*_G1_ = −2.2 V, *V*_B_ = 0.8 V, and *V*_G2_ = −2.3 V.

The oscillators were designed and fabricated in the WIN 0.25 μm GaN/SiC HEMT process. Figure 18 shows the micrograph of the second oscillator with a chip area of 2 × 1 mm^2^, including all test pads and dummy metal. Two center-tapped and one symmetric inductor are used. The die was attached to the PCB using epoxy for characterization. Figure 19 shows measured output voltages; differential signals are measured for the balanced oscillator. Figure 20 shows the measured output spectrum of the fundamental signal at 3.81 GHz with an output power of −0.706 dBm.

**Figure 21:**
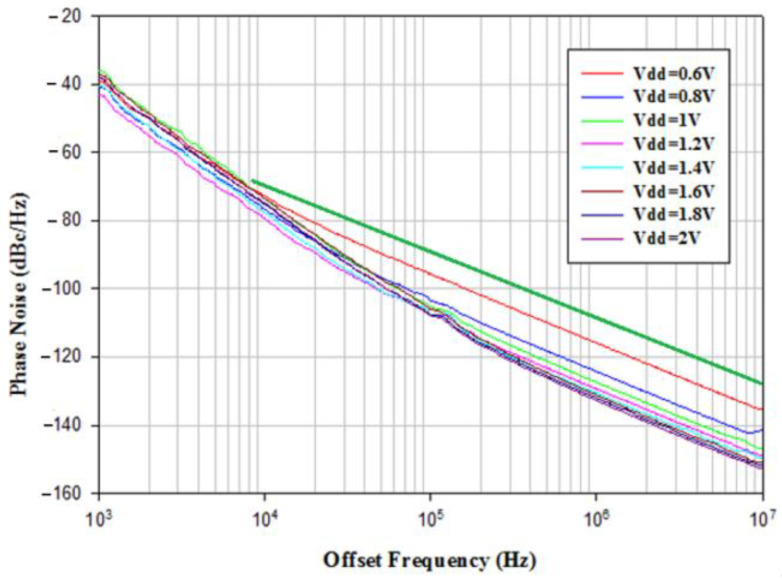
Measured phase noise. *V*_DD_ = 0.6–2 V, *V*_G1_ = −2.1 V, *V*_B_ = 0.8 V, and *V*_G2_ = −2.1 V. The green line is used as a guideline for phase noise due to thermal noise.

**Figure 22:**
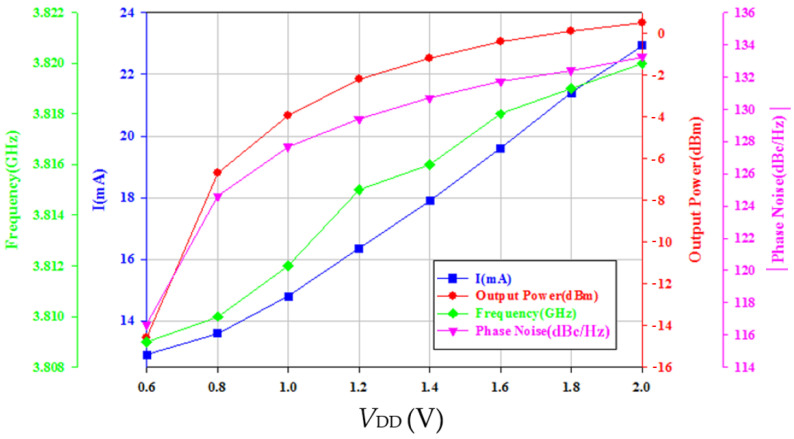
Measured frequency, phase noise, output power, and current consumption vs. *V*_DD_. *V*_DD_ = 0.6~2 V, *V*_G1_ = −2.2 V, *V*_B_ = 0.8 V, and *V*_G2_ = −2.3 V.

**Figure 23:**
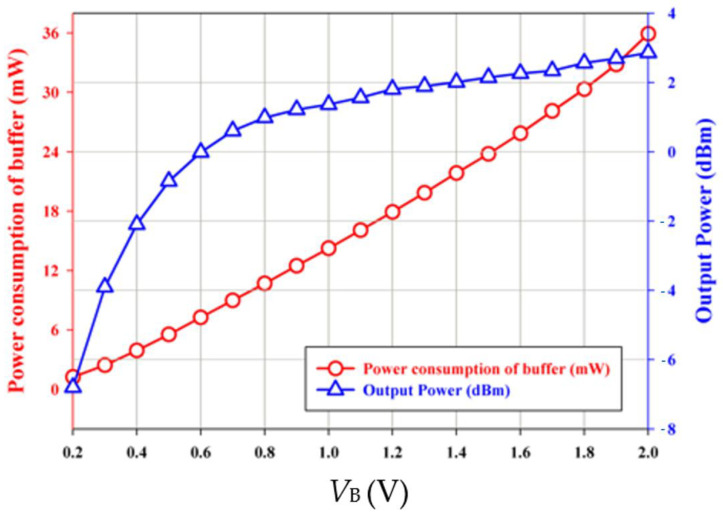
Measured power consumption and output power of the buffer versus *V*_B_. *V*_DD_ = 1.4 V, *V*_G1_ = −2.1 V, *V*_B_ = 0.2 ~ 2 V, and *V*_G2_ = −2.3 V.

To investigate the effect of varying supply bias on the phase noise of the balanced oscillator, the gate bias is fixed at −2.2 V and the supply bias is changed from 0.6 V to 2 V. Figure 21 shows the phase noise performance of VCO biased at *V*_DD_ = 0.6 V~2 V. At *V*_DD_ = 1.6 V, the power consumption is 31.36 mW, the measured phase noise of oscillator is about −131.73 dBc/Hz at 1 MHz offset frequency from 3.818 GHz oscillation frequency and has a slope of −20 dB/dec in the high frequency offset. The corner frequency increases as *V*_DD_ increases. By the calculation, the FOM is −188.4 dBc/Hz. Figure 22 shows measured frequency, phase noise at 1 MHz offset, output power, and current consumption versus *V*_DD_. As versus *V*_DD_ increases, the phase noise decreases, the oscillation frequency slightly increases, and the output power increases. Figure 23 shows the measured power consumption and output power of the buffer versus *V*_B_. Power consumption and output power of the buffer increase with increasing *V*_B_ because of enhanced channel conductance. The thermal noise-related phase noise is more sensitive to the supply variation than the flicker noise-related phase noise. Increasing the supply voltage is an effective way to reduce phase noise at high offset frequencies at the cost of power. The phase noise associated with thermal noise reduces as *V*_DD_ increases, while the phase noise at low offset frequency is not sensitive to supply bias; the latter is owing to device flicker noise. Table 1 is the performance comparison.

### 3.3. High-Supply Voltage Effect

**Figure 24:**
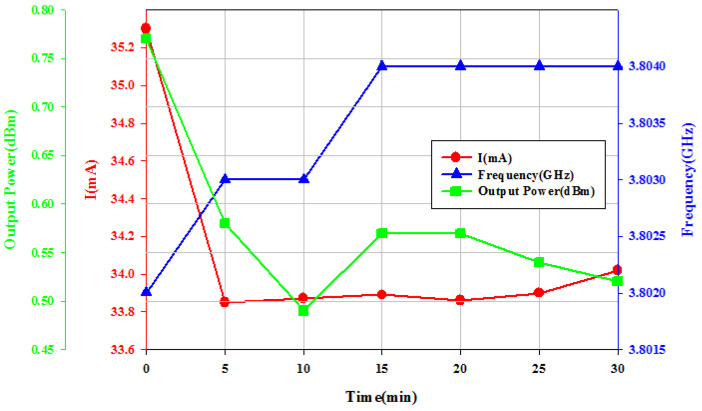
Measured output power, oscillation frequency, and core current consumption versus biased time. *V*_DD_ = 15 V, *V*_G1_= −2.1 V, *V*_B_ = 0.8 V, and *V*_G2_ = −2.1 V.

**Figure 25:**
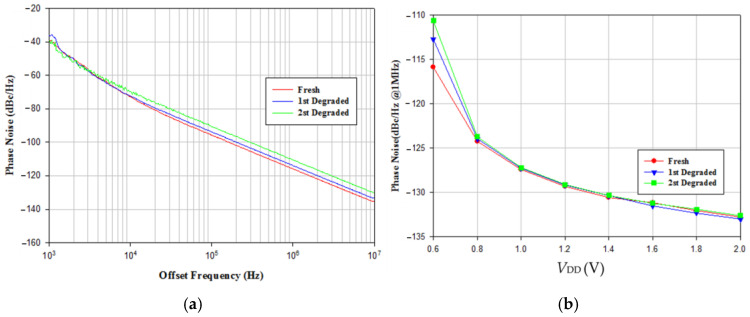
(**a**) Measured phase noises for *t*_bias_ = 0, 30, 60 min. *V*_DD_ = 0.6 V, *V*_G1_ = −2.1 V, *V*_B_ = 0.8 V, and *V*_G2_ = −2.1 V. (**b**) Measured post-stress phase noises at 1 MHz offset frequency. *V*_DD_ = 0.6–2 V, *V*_G1_ = −2.1 V, *V*_B_ = 0.8 V, and *V*_G2_ = −2.1 V.

The 2nd experiment was carried out by biasing the buffer at low supply and biasing the oscillator-core at *V*_DD_ = 15 V for 30 min; the output power and consumption decrease versus bias time, *t*_bias_, as shown in Figure 24. Measured data shows the effect of the VCO-core subject to the high *V*_DD_ bias. The hot-carrier stress causes degradation in oscillator parameters. Figure 25a shows measured phase noise after the stress time for *t*_bias_= 0, 30, and 60 min. At 1 MHz offset frequency, the phase noise increases with bias time. Figure 25b shows measured phase noise at a 1 MHz offset frequency; the degradation occurs at low supply voltage.

## 4. GaN HEMT Oscillator with Implicit-Resonator

Depending on the RF applications, the GaN oscillator can be classified as either a voltage-controlled oscillator (VCO) or a fixed-frequency oscillator (FFO) [20]. This section designs a third GaN HEMT cross-coupled FFO using a two-turn 8-shaped inductor to reduce the magnetic coupling noise and a degenerated-drain inductor to reduce the phase noise. The 8-shaped inductors are configured with 1-turn [21], 2-turn [22], 3-turn, and 4-turn structures and have not been used in GaN circuits.

### 4.1. Circuit Design

**Figure 26:**
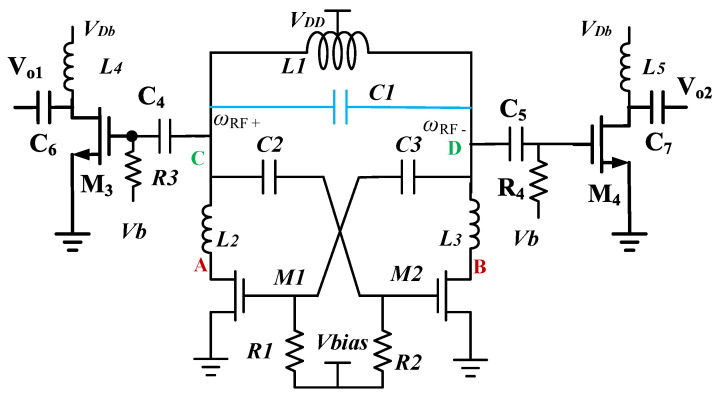
Circuit schematic of the third designed GaN FFO.

Figure 26 shows the FFO schematic. The FFO is designed using the WIN 0.12 μm RF GaN on SiC technology. Two 1-turn 8-shaped inductors (*L*_2_, *L*_3_) could improve the tank *Q*-factor, while (*M*_1_, *M*_2_) show channel resistance operating in the linear regime. Two-turn eight-shaped *L*_1_, capacitor *C*_1_, and parasitic capacitors (*C*_v1_, *C*_v2_) of HEMTs (*M*_1_, *M*_2_) resonate at the fundamental carrier. *V*_DD_ is the supply, and *V*_Bias_ is the gate bias. The three-and-a-half-turn spiral inductors (*L*_4_, *L*_5_) are buffer loads using a bias circuit (*C*_4_, *R*_3_). The pair (*M*_1_, *M*_2_) provides the negative differential resistance, compensating for the loss of the *LC* tank.

**Figure 27:**
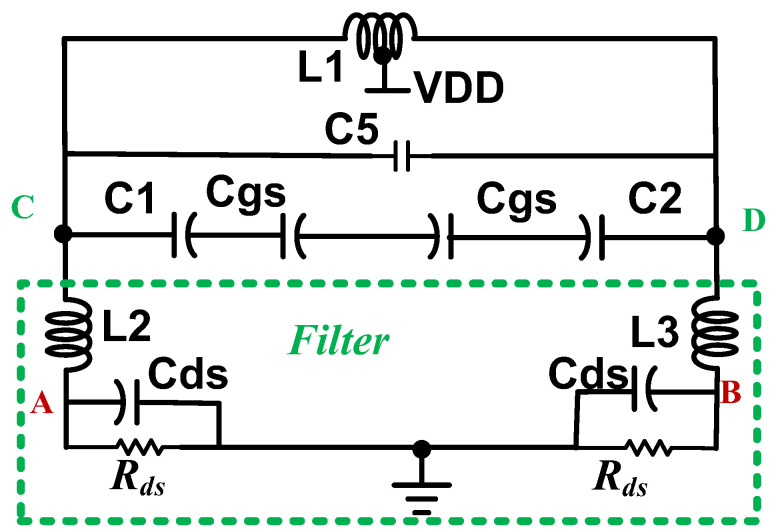
Equivalent circuit for the dual resonant network.

Figure 27 shows the equivalent circuit of Figure 26 with the dual resonant network by replacing *M*_1_ with the drain-source capacitor *C*_ds_ and channel resistor *R*_ds_. *L*_2_ in series with *C*_ds_ forms the harmonic resonator. Neglecting *R*_ds_, *C*_1_, and *C*_gs_ for simplicity, the two resonant frequencies are

(12) $$\omega^{2}=\frac{C_{5}L_{1}+L_{2}C_{ds}+0.5L_{1}C_{ds}}{2C_{5}L_{1}L_{2}C_{ds}}\pm \frac{\sqrt{{\left[C_{5}L_{1}+L_{2}C_{ds}+0.5L_{1}C_{ds}\right]}^{2}-4C_{5}L_{1}L_{2}C_{ds}}}{2C_{5}L_{1}L_{2}C_{ds}}$$

From the nodes A and B, *C*_5_ and (*L*_2_, *L*_3_) form the filter effect. *C*_ds_, *C*_ds_, and (*L*_2_, *L*_3_) form the harmonic resonance. The impedance peak seen between the nodes A and B is larger than the impedance peak seen between the nodes C and D. This is caused by (*L*_2_, *L*_3_).

**Figure 28:**
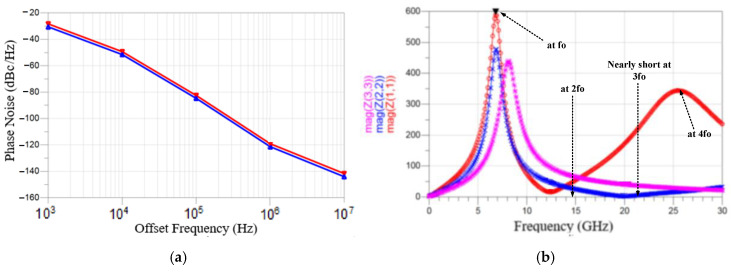
(**a**) Simulated phase noise. Red: deleted (*L*_2_, *L*_3_). (**b**) Impedances between A and B nodes. Purple/Red tank impedance with/without (*L*_2_, *L*_3_). Blue impedance between C and D nodes.

Figure 28a shows the simulated phase noise. Inductors (*L*_2_, *L*_3_) reduce the phase noise. The simulated phase noise is −119.3 dBc/Hz (−121.5 dBc/Hz) without (with) drain inductor. Figure 28b shows the three simulated tank impedances. The differential-mode (DM) impedance in purple color is from a reference VCO shown in Figure 26 without (*L*_2_, *L*_3_). It has one peak impedance. For the designed circuit, the DM impedance seen from the A and B nodes has two peaks, indicated in red color. The fundamental DM peak impedance occurs at 7.3 GHz, and the 3rd harmonic DM impedance can reduce the phase noise by reducing the Groszkowski frequency shift [23]. It also shows an impedance dip at the second harmonic. The dip filters out the second harmonic, and it reduces the phase noise. The first peak is caused by (*L*_1_, *L*_2_, *L*_3_) in shunt with (*C*_ds_, *C*_ds_). The impedance dip is caused by (*L*_2_, *L*_3_) in series with *C*_5_. The high-frequency impedance peak is caused by (*L*_2_, *L*_3_) in shunt with (*C*_ds_, *C*_ds_). For the designed circuit, the DM impedance seen between the C and D nodes has one peak, indicated in blue color. It is smaller than the reference one, except for the peak. This will reduce the harmonics. Inductors (*L*_2_, *L*_3_) shift the peak impedance to a lower frequency.

**Figure 29:**
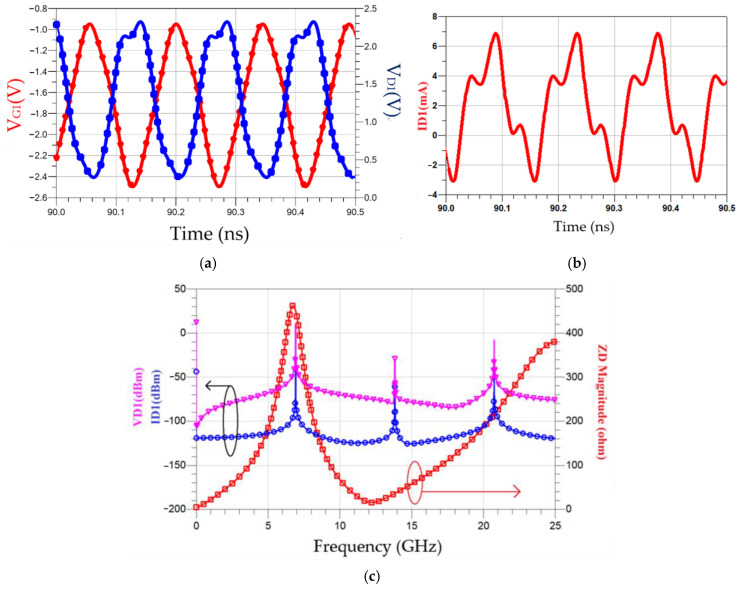
Simulated waveforms and frequency-domain analysis of the oscillator: (**a**) drain (V_D_) and gate (V_G_) voltage waveforms of transistor *M*_1_; (**b**) drain current (I_D_) waveform of transistor *M*_1_; (**c**) DFT results of drain voltage (V_D_), drain current (I_D_), and impedance magnitude between nodes A and B.

Using the circuit schematic depicted in Figure 26, simulation analyses were performed at nodes A and B to obtain the waveforms shown in Figure 29. As illustrated in Figure 29a, the drain voltage (V_D1_) exhibits rapid transitions between on and off states. Figure 29b shows that the drain current I_D1_ contains harmonics. Figure 29c compares the impedance magnitude between nodes A and B with the DFT results of V_D1_ and I_D1_. It demonstrates higher impedance magnitudes at the fundamental and third harmonic frequencies compared to the second harmonic. The dip reduces the second harmonic.

**Figure 30:**
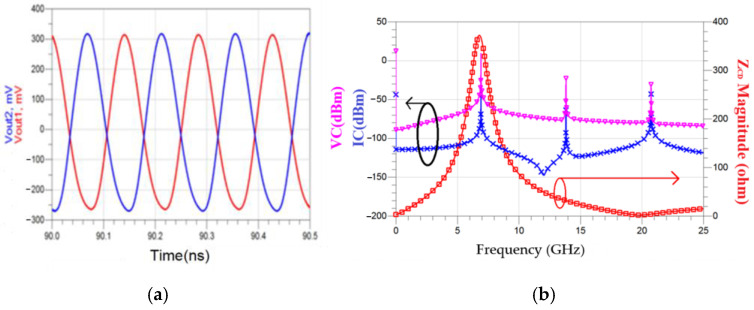
Simulated drain voltage *V*_out1_ waveform (**a**,**b**), DFT analysis of voltage (VC) and current (IC) at node C, and the impedance magnitude between nodes C and D (Z_CD_), based on the oscillator schematic in Figure 26. *V*_DD_ = 1.3 V, *V*_bias_ = −1.6 V, *V*_DB_ = 5 V, and *V*_B_ = −2 V.

**Figure 31:**
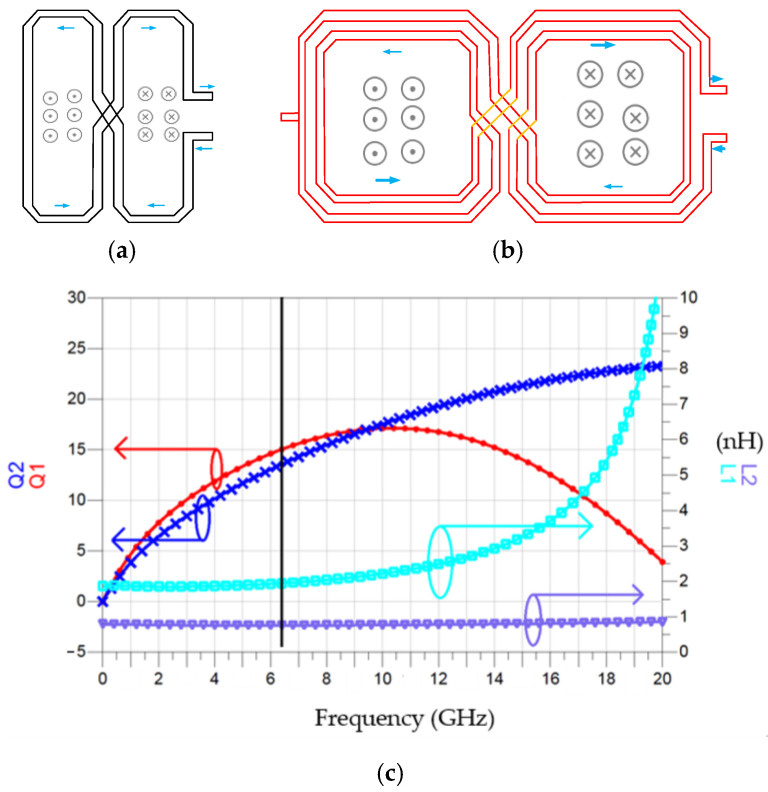
(**a**) Layout of *L*_2_. (**b**) Layout of *L*_1_. (**c**) Simulated inductance and *Q*-factor.

Figure 30 shows the simulated time-domain drain voltage *V*_out1_ waveform and DFT. The carrier is at 6.971 GHz and has an output power of −1.81 dBm. The second harmonic has an output power of −24.492 dBm. Figure 31a shows the layout of a one-turn series twisted eight-shaped inductor, and Figure 31b shows a two-turn eight-shaped inductor consisting of two 2-turn O-shaped inductors in a twisted series. Figure 31c shows the simulated inductance and *Q*-factor of the 8-shaped inductor. At 6.4 GHz, the inductance of *L*_2_ is 0.758 nH, and the *Q*-factor is 13.568. The inductance of *L*_1_ is 1.947 nH, and the *Q*-factor is 15.043.

### 4.2. Measurement and Discussion

**Figure 32:**
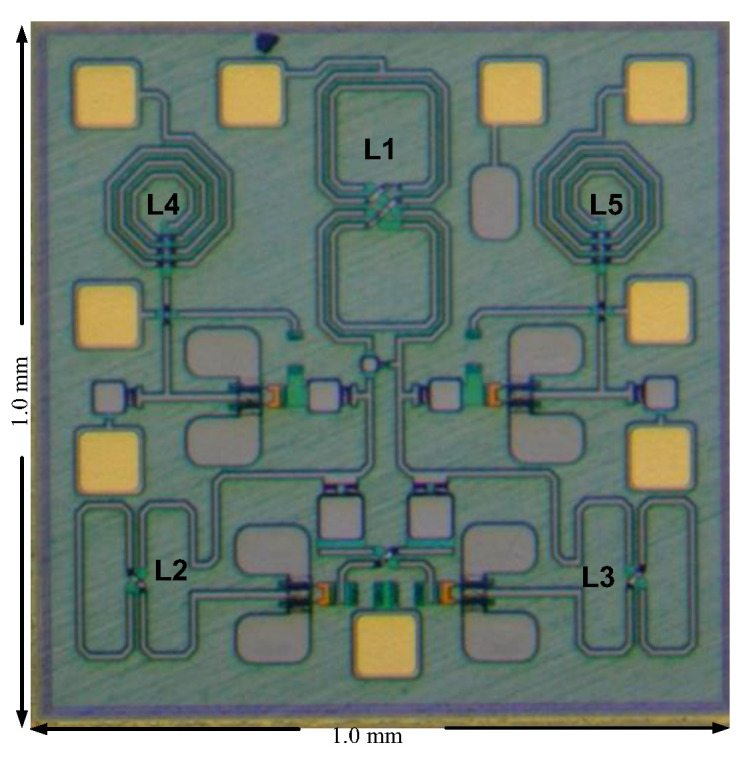
Chip photo of FFO.

**Figure 33:**
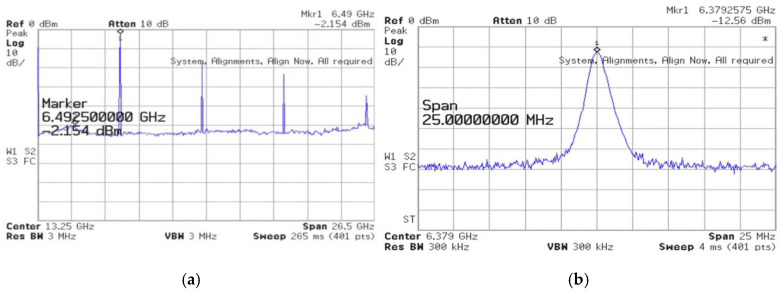
Measured (**a**) full-span spectrum and (**b**) expanded spectrum of the VCO. *V*_DD_ = 1.3 V, *V*_bias_ = −1.6 V, *V*_DB_ = 5 V, and *V*_B_ = −2 V.

**Figure 34:**
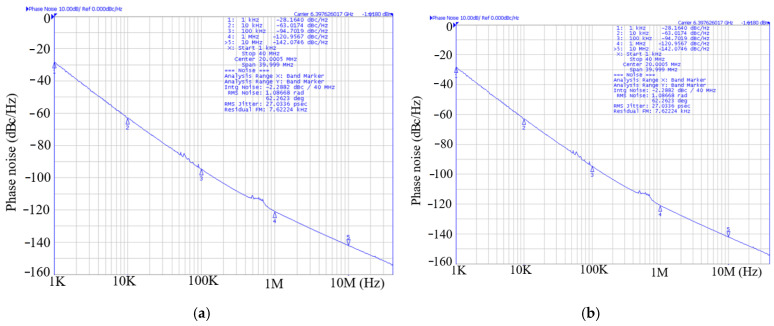
(**a**) Measured phase noise of the GaN FFO. *V*_DD_ = 1 V, *V*_bias_= −1.6 V, *V*_DB_ = 5 V, and *V*_B_ = −2 V. (**b**). Measured phase noise of the GaN FFO. *V*_DD_ = 1.3 V, *V*_bias_ = −1.6 V, *V*_DB_ = 5 V, and *V*_B_ = −2 V.

**Figure 35:**
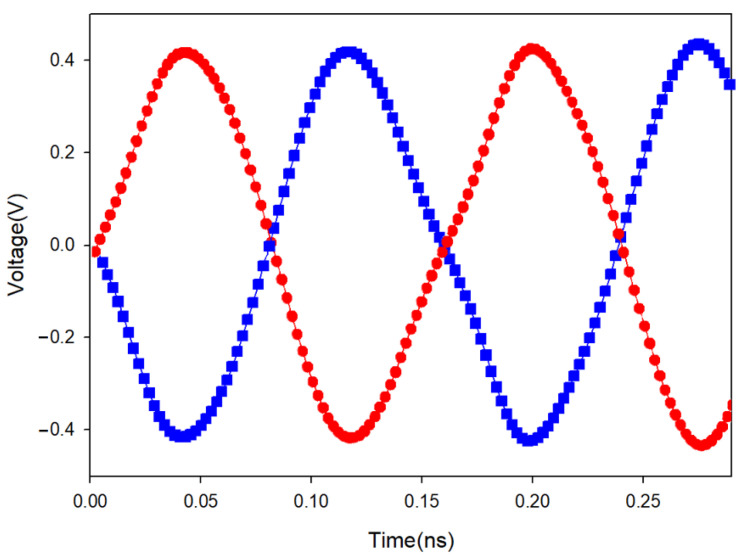
Measured output voltages of the GaN FFO. *V*_DD_ = 1.3 V, V_DB_ = 5 V, V_B_ = −2 V, and V_bias_ = −1.6 V.

The 3rd FFO has been designed and fabricated in the WIN 0.12 μm GaN technology. The FFO was measured on the PC board. The measurements of oscillation frequency were conducted with the Keysight Spectrum Analyzer, while the phase noise was measured using the Keysight signal source analyzer. As shown in Figure 32, the die micrograph occupies an area of 0.8 × 0.8 mm^2^, including all test pads and dummy metal. Figure 33 shows the measured spectrum of the GaN FFO at 6.49 GHz. The carrier output power is −2.154 dBm. The second harmonic output power is −17.64 dBm, and the 3rd harmonic output power is −23.27 dBm. Figure 34a shows the measured phase noise of the FFO at *V*_DD_ = 1 V. The phase noise at 1 MHz offset from the carrier at 6.397 GHz is −120.9 dBc/Hz. The corner frequency is 800 KHz. The FOM is −188.81 dBc/Hz. Figure 34b shows the measured phase noise of the FFO at *V*_DD_ = 1.3 V. The phase noise at 1 MHz offset from the carrier at 6.397 GHz is −123.688 dBc/Hz. The corner frequency is 1 MHz. At 6.3971 GHz, the calculated FOM is −190.09 dBc/Hz. Similar to Figure 30a, Figure 35 shows the measured output voltages of the GaN FFO. Figure 33a resembles Figure 30b; the *M*_1_ and *M*_2_ cause the harmonic. Table 1 is the performance comparison of GaN oscillators.

## 5. Conclusions

This paper designs three GaN HEMT oscillators with a buffer. The oscillators include a single-ended oscillator, a balanced oscillator, and a cross-coupled oscillator. Two biases, oscillator supply and buffer supply, are used to optimize the oscillator performance and reliability of the circuit design. The first 8.86 GHz oscillator consists of an oscillator core biased at a supply voltage of 0.8 V and a buffer biased at 3 V. It can supply large output power because of a buffer biased at a high supply voltage, and it achieves low phase noise by operating the reliable oscillator core at a low supply voltage. The FoM of this oscillator is −199.8 dBc/Hz. We also study the hot-carrier stress effect on the 8.9 GHz GaN oscillator. When the core oscillator is subject to a supply voltage of 8 V, the post-bias oscillator phase noise measured at low supply voltage degrades. High FFO-core supply bias degrades the FOM. Biasing the oscillator core at low supply and the buffer at high supply is a good operation condition because high-oscillator FOM is maintained. The second 3.8 GHz balanced oscillator consists of two feedback sub-oscillators in a balanced configuration by suppressing the common mode oscillation, and the oscillator feedback uses a differential left-handed transmission line *LC* network for the first time in GaN HEMT oscillators. The balanced oscillator provides a differential signal, as verified by the time domain waveform. The output power increases and the phase noise improves with the supply voltage. For the present design, the output power can be controlled by buffer bias for higher output power. The oscillator phase noise degrades with high oscillator-core stress supply operation. The third 6.39 GHz C-band *LC*-tank oscillator with a buffer biased at high supply and the FFO-core biased at low supply is based on 0.12 μm WIN GaN-on-SiC technology. The oscillator uses 8-shaped inductors for suppressing magnetic coupling noise. At the supply of 1.3 V, the phase noise of the oscillator consists of 1/f^2^ and 1/f^3^ noise; the FFO FOM is −190.09 dBc/Hz. The FFO using two 1-turn 8-shaped inductors improves the oscillator phase noise by forming an intrinsic *LC* resonator to null the effect of the 2nd harmonic current on the phase noise.

## Figures and Tables

**Table 1:** Performance comparison of GaN oscillators.

Ref	Proc (um)	Topol	Vdd (V)/ Pdis (mW)	fo GHz	PN dBc/Hz	FOM dBc/Hz	8-Shaped Inductor
[7]	0.25	Hartley	28/1456	7.9	−112 *	−178	no
[10]	0.25	Balanced Colpitts	6/180	9.92	−136	−193	no
[14]	-	Push-push	15/600	9.1	−130	−181	no
[15]	0.25	Common source	10/600	9.9	−135	−187	no
[16]	0.25	Common gate	30/10625	9.55	−115.0	−154.0	no
[17]	0.25	Cross-coup	0.4/2.669	4.746	−121.77	−191.03	no
[18]	0.25	Bal Colpitts Osc	15/600	8.6	−102	−172.9	no
[19]	0.25	Cross-Coupled VCO	-/747	23.9–24.4	−109.4	−168.3	no
[20]	-	Osc (on-board)	28/4.9	2.44	−123.1	−164.03	no
This ^1^	0.25	feedback	0.8/2.45	8.86	−124.8	−199.8	no
This ^2^	0.25	LH feedback	1.6/31.36	3.818	−131.73	−188.4	no
This ^3^	0.12	Cross-Coupled Osc	1.3/9.373	6.397	−123.688	−190.09	yes

* @100 KHz. ^1^ chip in Figure 2. ^2^ chip in Figure 18. ^3^ chip in Figure 32.

## Data Availability

The original contributions presented in this study are included in the article. Further inquiries can be directed to the corresponding author.
